# A missense mutation in *Katnal1* underlies behavioural, neurological and ciliary anomalies

**DOI:** 10.1038/mp.2017.54

**Published:** 2017-04-04

**Authors:** G Banks, G Lassi, A Hoerder-Suabedissen, F Tinarelli, M M Simon, A Wilcox, P Lau, T N Lawson, S Johnson, A Rutman, M Sweeting, J E Chesham, A R Barnard, N Horner, H Westerberg, L B Smith, Z Molnár, M H Hastings, R A Hirst, V Tucci, P M Nolan

**Affiliations:** 1 MRC Harwell Institute, Harwell Science and Innovation Campus, Didcot, UK; 2 Department of Neuroscience and Brain Technologies, Istituto Italiano di Tecnologia, Genoa, Italy; 3 Department of Physiology, Anatomy and Genetics, University of Oxford, Oxford, UK; 4 Centre for PCD Diagnosis and Research, Department of Infection, Immunity and Inflammation, RK Clinical Sciences Building, University of Leicester, Leicester, UK; 5 MRC Laboratory of Molecular Biology, Cambridge Biomedical Campus, Cambridge, UK; 6 MRC Centre for Reproductive Health, University of Edinburgh, The Queen’s Medical Research Institute, Edinburgh, UK

## Abstract

Microtubule severing enzymes implement a diverse range of tissue-specific molecular functions throughout development and into adulthood. Although microtubule severing is fundamental to many dynamic neural processes, little is known regarding the role of the family member Katanin p60 subunit A-like 1, KATNAL1, in central nervous system (CNS) function. Recent studies reporting that microdeletions incorporating the *KATNAL1* locus in humans result in intellectual disability and microcephaly suggest that KATNAL1 may play a prominent role in the CNS; however, such associations lack the functional data required to highlight potential mechanisms which link the gene to disease symptoms. Here we identify and characterise a mouse line carrying a loss of function allele in *Katnal1*. We show that mutants express behavioural deficits including in circadian rhythms, sleep, anxiety and learning/memory. Furthermore, in the brains of *Katnal1* mutant mice we reveal numerous morphological abnormalities and defects in neuronal migration and morphology. Furthermore we demonstrate defects in the motile cilia of the ventricular ependymal cells of mutants, suggesting a role for *Katnal1* in the development of ciliary function. We believe the data we present here are the first to associate KATNAL1 with such phenotypes, demonstrating that the protein plays keys roles in a number of processes integral to the development of neuronal function and behaviour.

## Introduction

Microtubule severing enzymes are a family of AAA-ATPase proteins that participate in fundamental cellular processes such as mitosis, ciliary biogenesis and growth cone motility. In neurons this family is known to control such processes as axonal elongation^1^ and synaptic development.^2^ In addition, mutations in microtubule severing enzyme genes *SPG4*, *KATNB1* and *KATNAL2* are associated with hereditary spastic paraplegia, cerebral malformations and autism, respectively,^3, 4, 5, 6^ and mutations in *Fign* cause a range of phenotypes in mice.^7^ Currently the microtubule severing enzyme KATNAL1 is poorly characterised and it is not yet understood how the enzyme functions in the nervous system. Recent evidence from genetic characterisation of human patients suggests that haploinsufficiency of *KATNAL1* is linked with a number of symptoms including intellectual disability (ID) and craniofacial dysmorphologies.^8, 9^ It is also notable that a very rare *KATNAL1* mutation has been associated with schizophrenia^10^ (http://atgu.mgh.harvard.edu/~spurcell/genebook/genebook.cgi?user=guest&cmd=verb-gene&tbox=KATNAL1) and that Peters syndrome and autism have both been associated with the chromosomal region containing the *KATNAL1* locus.^11, 12^ Although such association studies strongly suggest that *KATNAL1* plays a fundamental role in the central nervous system (CNS), additional studies using cellular or animals models are required to understand how the gene may be causative for disease. Here we present the first study describing neural and behavioural deficits associated with a loss of function allele of *Katnal1* in the mouse. This mutant mouse line was independently identified in two parallel phenotyping screens, which demonstrated that mutant mice showed both male sterility and circadian phenotypes. Subsequent behavioural investigations demonstrated that this mutation is associated with anxiety and memory deficits. Underlying these behavioural phenotypes, we identified histopathological abnormalities in the brains of *Katnal1*^*1H/1H*^ mutants, including disordered cellular layers in the hippocampus and cortex and substantially larger ventricles. Further investigations demonstrated that *Katnal1*^*1H/1H*^ mice show neuronal migration and ciliary function deficits suggesting KATNAL1 plays an essential role in these processes. These findings are the first to our knowledge to conclusively show that mutations in *Katnal1* lead to behavioural and neuronal disturbances and provide insight regarding the clinical associations that have been linked to the gene.

## Materials and methods

### Animals

Animal studies were performed under guidance from the Medical Research Council in Responsibility in the Use of Animals for Medical Research (July 1993) and Home Office Project Licences 30/2686 and 80/2310 (UK) and project licence 106/2009-B (Italy). ENU mutagenesis and animal breeding regimes were performed as previously described.^13^ Phenotyping was performed on mouse cohorts that were partially or completely congenic on the C57BL/6 J background.

### Circadian wheel running

Circadian wheel running was performed as previously described.^14^

### Sleep assessment by electroencephalography and electromyography

Electroencephalography and electromyography was performed as previously described.^15^

### Behavioural phenotyping

#### Spontaneous alternation

Mice were placed in a walled T-maze (black polyvinyl chloride, lined with sawdust; stem=88 × 13 cm; arms=32 × 13 cm) and allowed to enter a goal arm of their choice. The mouse was confined in the goal arm for 30 s, before being allowed a second free choice of goal arm. An alternation was recorded if the second choice differed from that of the first. One trial was performed per day for 10 days.

#### Open field behaviour

Mice were placed into a walled arena (grey polyvinyl chloride; 45 × 45 cm) and allowed to explore for 20 min. Animals were monitored by EthoVision XT analysis software (Noldus, Wageningen, Netherlands).

#### Video tracking in the home cage

Activity in the home cage was recorded by video tracking as previously described.^16^

#### Morris water maze and ultrasonic vocalisation

These tests were performed as previously described.^17^

### Brain histology and immunofluorescence

Brains were mounted in OCT (VWR) and 12 μm coronal sections taken. Sections were stained with hematoxylin and eosin, or immunolabelled following standard protocols.

### Neuronal migration assessment

*In vivo* neuronal migration assessment was performed as previously described^18^ using embryos at either E13 or E15 (three mothers per age group) and pups at P9. Cell counts were performed using ImageJ (NIH, Bethesda, MD, USA).

*In vitro* neuronal migration assessment was performed using a Boyden chamber migration protocol as previously described.^19^

### Micro-computed tomography scanning

Micro-computed tomography was performed using a Skyscan 1172 at 90 kV, 112 μA using an aluminium and copper filter, a rotation step of 0.250 degrees and a pixel size of 4.96 μm.

Segmentation, volume calculation and 3D modelling was performed using ITK-SNAP version 3.0.0 (ref. 20) and 3DSlicer.^21^

### Golgi-Cox staining of neurons

Golgi-Cox neuronal staining was performed using the FD Rapid GolgiStain Kit (FD NeuroTechnologies, Columbia, MD, USA). Neurons were analysed using ImageJ.

### Motile cilia analysis

Brains from P2 mice were dissected, and the dorsal cerebral half was sectioned (250 μm) through the floor of the lateral and 3rd ventricle using a vibratome. Ciliary beat frequency and pattern was analysed as previously described.^22^

### Electron microscopy

For Scanning Electron Microscopy the ependymal lining of the lateral ventricle was fixed in 2.5% glutaraldehyde, 2% paraformaldehyde in 0.1 m phosphate buffer, incubated in 2% osmium tetroxide, and dehydrated through ethanol solutions. Samples were critical point dried using an Emitech K850 (Quorum Technologies, East Sussex, UK), coated with platinum using a Quorom Q150R S sputter coater (Quorum Technologies). and visualised using a JEOL LSM-6010 scanning electron microscope (Jeol, Herts, UK).

Transmission electron microscopy was performed as previously described.^22^

### Statistical analysis

Data was analysed using two-tailed students T test or AVOVA using SPSS (IBM) or GraphPad Prism 5.0 (GraphPad Software, La Jolla, CA, USA). Significance level for all analysis was set at *P*<0.05. All graphs are presented showing mean±s.e.m.

Additional and more detailed methods can be found in supplementary information.

## Results

### Identification and cloning of the *Katnal1*^*1H*^ mutation

To identify novel gene mutations affecting circadian behaviour we undertook a circadian running wheel screen of pedigrees of *N*-ethyl-*N*-nitrosourea mutagenised mice.^13^ In one pedigree 17.65% of animals showed a short circadian period in constant darkness (<23 h observed in 12 out of 68 animals screened). An outcross using an affected female produced no affected animals (33 animals screened). In subsequent intercross screens 15.5% of animals were affected (53 out of 342 animals screened), suggesting that the pedigree carries a mutation causing a recessive circadian phenotype which is 60% penetrant. We found no gender bias in affected animals (proportion of affected animals: male=47.2% female=52.8%).

Concurrently a male sterility phenotype was identified within the same pedigree.^23^ Genome-wide SNP linkage analysis mapped the circadian and sterility phenotypes to the same region on chromosome 5 and subsequent sequencing identified the causative mutation as a T to G single point mutation within exon seven of the Katnal1 gene. For full details of mapping and identification of the mutation see reference 23. This mutant allele was designated *Katnal1*^*1H*^, and results in a leucine to valine substitution at residue 286 of the protein. *In vitro* functional analysis demonstrated that the mutation is a recessive loss-of-function allele.^23^ 3D modelling of the protein suggests that this loss of function is due to hydrophobic changes in the AAA domain of the enzyme (Supplementary Figure S1). Genotyping confirmed that the mutation was homozygous in affected circadian animals and wild type or heterozygous in unaffected animals, confirming that *Katnal1*^*1H*^ was causative for the circadian phenotype.

### Circadian and sleep anomalies in *Katnal1*^*1H/1H*^ mice

**Figure 1:**
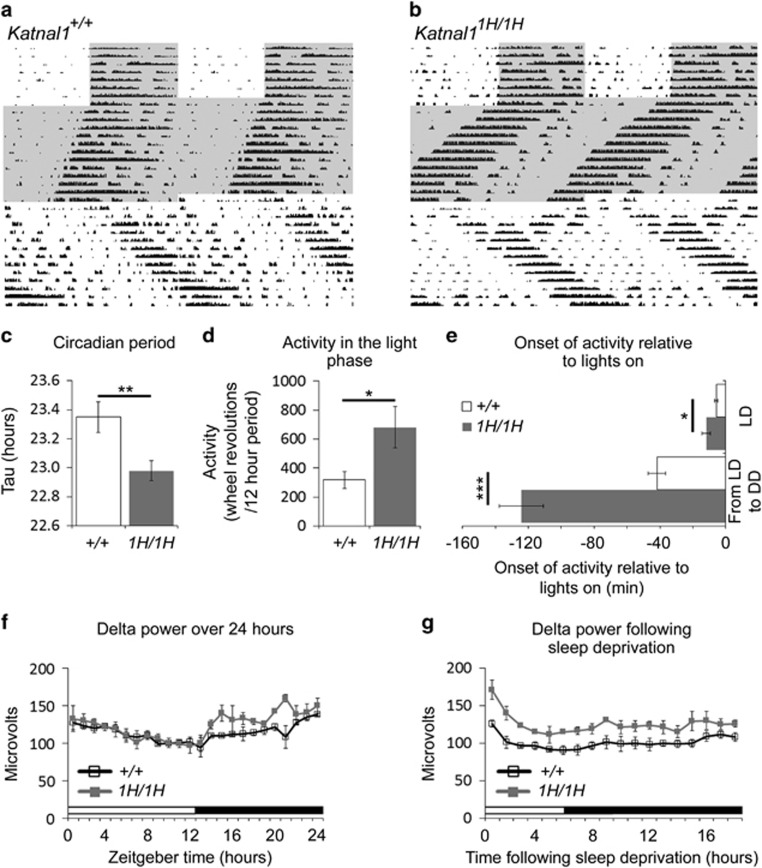
Circadian and sleep anomalies in *Katnal1*^*1H/1H*^ mice. (**a** and **b**): Double plotted actograms from wild-type (**a**) and *Katnal1*^*1H/1H*^ (**b**) animals. Wheel running activity is represented by vertical black bars, with each horizontal row representing two days of time; shaded regions show time spent in darkness, unshaded regions show time spent in light (see reference 14 for descriptions of double plotted actograms). Compared to wild-type littermates, *Katnal1*^*1H/1H*^ animals have a shorter period (**c**), are more active in the light phase of the light/dark cycle (**d**) and show an earlier onset of activity in light/dark transitions and in the transition from light/dark cycles to constant darkness (**e**). In EEG recordings during sleep, *Katnal1*^*1H/1H*^ mice show increased non-REM delta power in the dark phase of the light/dark cycle (**f**) and following sleep deprivation (**g**). *P⩽0.05; **P⩽0.01; ***P⩽0.001. EEG, electroencephalography; DD, constant darkness; LD, light/dark cycle.

More extensive circadian phenotyping conducted on *Katnal1* homozygotes (*Katnal1*^*1H/1H*^) and wild-type littermates (*Katnal1*^*+/+*^) confirmed that *Katnal1*^*1H/1H*^ mice had a shorter free-running circadian period (Figures 1a–c) and furthermore revealed that *Katnal1*^*1H/1H*^ animals were more active in the light phase of the light/dark cycle (Figure 1d), showed increased anticipation of light to dark transitions and greater shift in activity onset when released from light/dark cycles to constant darkness (Figure 1e). Data and cohort details are given in Supplementary Table S1. Bioluminescence recordings performed using PER2::LUCIFERASE reporter mice carrying the *Katnal1*^*1H*^ mutation revealed that these circadian changes were not due to changes to the core molecular clock of the suprachiasmatic nucleus (the site of the master circadian clock in the brain; Supplementary Figure S2).

Circadian disruptions are often associated with deficits in sleep homeostasis. Therefore to complement our circadian studies we conducted wireless electroencephalography recordings over a baseline period of 24 h and following a 6 h period of sleep deprivation. A detailed summary of electroencephalography analysis is given in Supplementary Table S1. Compared to wild-type littermates, the non-REM delta power of *Katnal1*^*1H/1H*^ mice was higher in the dark phase of baseline sleep (mixed ANOVA, interaction factors ‘genotype X time, F(1,88)=8.91, *P*=0.0175) (Figure 1f) and in both the light and dark phases of recovery sleep (mixed ANOVA, interaction factors ‘genotype X time’, F(1,136)=11.93, *P*=0.0086; Figure 1g). All other sleep parameters were unaffected in *Katnal1*^*1H/1H*^ animals.

### *Katnal1*^*1H/1H*^ mice display a spectrum of behavioural deficits

Human patients carrying a heterozygous deletion incorporating the *Katnal1* locus show a number of cognitive deficits including ID and a delay in language acquisition.^8, 9^ We therefore investigated whether these deficits were modelled in *Katnal1*^*1H/1H*^ mice by subjecting animal cohorts to a battery of behavioural tests. Data and cohort details are given in Supplementary Table S2.

**Figure 2:**
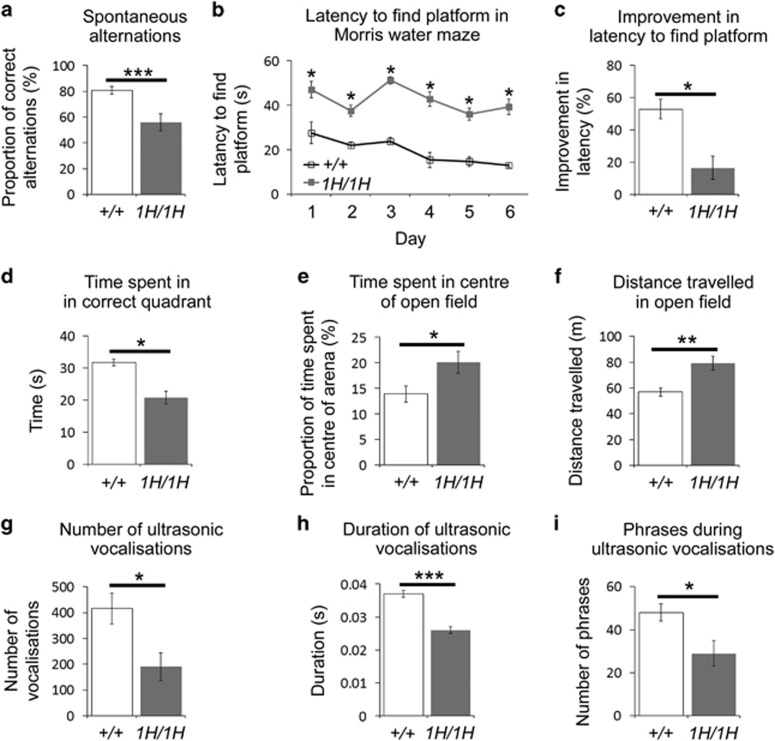
*Katnal1*^*1H/1H*^ mice display a spectrum of abnormal behaviours. Compared to wild-type littermates, *Katnal1*^*1H/1H*^ mice show: reduced spontaneous alternations in a T-maze (**a**); an increased latency to find the platform in Morris water maze trials (**b**); reduced improvement to find the platform in the Morris water maze (**c**); reduced time in the correct quadrant of the Morris water maze (**d**); increased time in the centre of an open field (**e**); greater movement in an open field (**f**); fewer USVs (**g**); shorter USVs (**h**); fewer phrases in their USVs (**i**). *P⩽0.05; **P⩽0.01; ***P⩽0.001. USV, ultrasonic vocalisation.

Both working memory and spatial memory were significantly poorer in *Katnal1*^*1H/1H*^ mice, as evidenced by reduced spontaneous alternations in a T-maze (Figure 2a) and in the Morris water maze where mutants take longer to find the platform in acquisition trials (Figure 2b), show poorer improvements in the latency to find the platform (Figure 2c) and spend less time in the target quadrant than wild types (Figure 2d). Anxiety and locomotor activity was assessed by monitoring activity in an open field arena. Here *Katnal1*^*1H/1H*^ animals spent a higher proportion of time in the centre of the arena (Figure 2e) and travelled over longer distances (Figure 2f) compared to wild-types. To differentiate between activity and anxiety changes in the open field arena we analysed activity in both the anxiogenic centre and the anxiolytic periphery of the arena. This analysis demonstrated that *Katnal1*^*1H/1H*^ animals showed increased activity in both regions of the open field (distance travelled in centre of open field: wild type=164±12 m, *Katnal1*^*1H/1H*^=243±20 m, *P*=0.02; distance travelled in periphery of open field: wild type=4.3±0.2 m, *Katnal1*^*1H/1H*^=6±0.3 m, *P*=0.004). Conversely when mouse activity was recorded in the home cage, we found no difference between genotypes (distance travelled over 24 h: wild type=399±77 m, *Katnal1*^*1H/1H*^=418±41 m, *P*=0.833) suggesting that the former activity differences were due to the novel environment of the open field rather than generalised hyperactivity in *Katnal1*^*1H/1H*^ animals. Finally, in certain conditions (such as maternal separation) mice emit ultrasonic vocalisations (USVs). To test whether *Katnal1*^*1H/1H*^ animals vocalised differently to wild types, we separated pups at postnatal days 7–8 (the age at which mice show peak of USV emission^24^) and recorded their USVs. In these tests, compared to wild types, *Katnal1*^*1H/1H*^ pups produced fewer (Figure 2g) and shorter (Figure 2h) vocalisations, containing fewer phrases (Figure 2i).

### Gross brain morphological abnormalities in *Katnal1*^*1H/1H*^ mice

Since we observed a number of behavioural phenotypes in *Katnal1*^*1H/1H*^ mice, we performed histological analysis to ascertain whether differences in brain histology underlied these behaviours. Data and cohort details are given in Supplementary Table S3.

**Figure 3:**
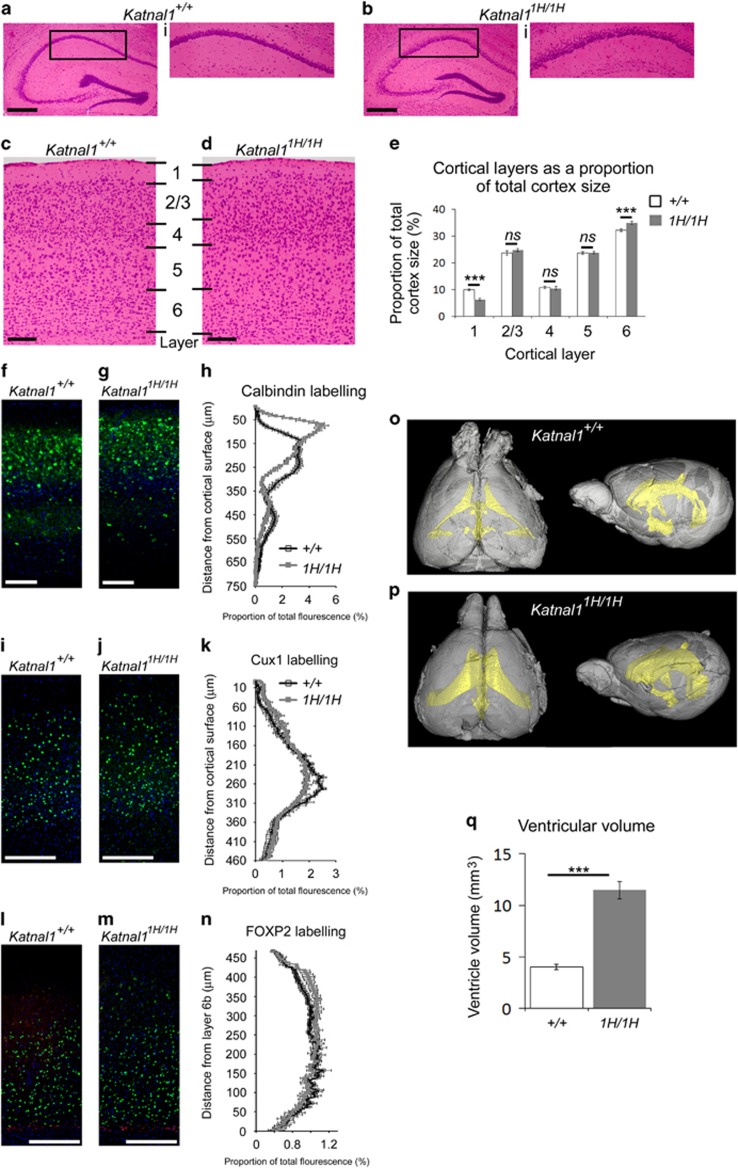
Aberrant brain histology in *Katnal1*^*1H/1H*^ mice. (**a**,**b**): Hippocampal histology in wild-type (**a**) and *Katnal1*^*1H/1H*^ (**b**) animals. Inserts (**i**) show CA1 layer. (**c**,**d**): Cortical layers in wild-type (**c**) and *Katnal1*^*1H/1H*^ (**d**) animals. (**e**: *Katnal1*^*1H/1H*^ animals have a narrower cortical layer 1 and a wider layer 6, compared to wild types. (**f** to **n**) Immunofluorescence of cortical layers: Calbindin immunofluorescence in cortical sections of wild-type (**f**) and *Katnal1*^*1H/1H*^ (**g**) animals. (**h**): Quantification of calbindin immunofluorescence demonstrates that *Katnal1*^*1H/1H*^ animals have a higher proportion of labelling towards the cortical surface than wild types. CUX1 immunofluorescence in cortical sections of wild-type (**i**) and *Katnal1*^*1H/1H*^ (**j**) animals. (**k**): Quantification of CUX1 immunofluorescence demonstrates that *Katnal1*^*1H/1H*^ animals have a higher proportion of labelling towards the cortical surface than wild types. FOXP2 (green) and CTGF (red) immunofluorescence in cortical sections of wild-type (**l**) and *Katnal1*^*1H/1H*^ (**m**) animals. (**n**): Quantification of FOXP2 immunofluorescence demonstrates that *Katnal1*^*1H/1H*^ animals have a higher proportion of labelling distant from layer 6b than wild-types. (**o**,**p**): μCT scans of the ventricular system (yellow) in wildwild-type (**o**) and *Katnal1*^*1H/1H*^ (**p**) brains. (**q**): Quantification of ventricular volume demonstrates that *Katnal1*^*1H/1H*^ mice have larger ventricles than wild types. Scale bars: 500 μm in **a** and **b**; 100 μm in **d**–**g**. ***P⩽0.001.

Analysis of hematoxylin and eosin stained brain sections revealed that, compared to wildtype littermates, *Katnal1*^*1H/1H*^ animals had less tightly packed pyramidal cell layers in the hippocampus (Figures 3a and b) and a narrower cortical layer 1 and wider cortical layer 6 (Figures 3c–e). To confirm these cortical layer differences, immunofluorescence was performed using the cortical layer markers calbindin (layers 2/3 and 5; Figures 3f and g), CUX1 (layers 2/3 and 4; Figures 3i and j), FOXP2 (layer 6a) and CTGF (layer 6b) (Figures 3l and m). Quantification of fluorescence intensity demonstrated that in *Katnal1*^*1H/1H*^ cortex both calbindin and CUX1 labelling was more intense closer to the cortical surface, which is consistent with the reduction in the size of layer 1 (two-way analysis of variance (ANOVA), interaction factors ‘genotype X distance of fluorescence from cortical surface’, calbindin: F(75,988)=16.8, *P*<0.0005; CUX1: F(93,372=2.17, *P*=0.001; Figures 3h and k). Similar quantification revealed that FOXP2 labelling extended further from layer 6b (as labelled by CTGF) in the *Katnal1*^*1H/1H*^ cortex, which is consistent with an increase in the size of layer 6 (two-way ANOVA, interaction factors ‘genotype X distance of fluorescence from CTGF labelling:’ F(93,372)=1.32, *P*=0.038; Figure 3n). Finally, three dimensional models of the ventricular system were constructed from brain micro-computed tomography scans (Figures 3o and p). Volumetric analysis revealed that *Katnal1*^*1H/1H*^ mice had substantially larger ventricles than wild types (Figure 3q).

### Neuronal migration and morphology defects in *Katnal1*^*1H/1H*^ brains

**Figure 4:**
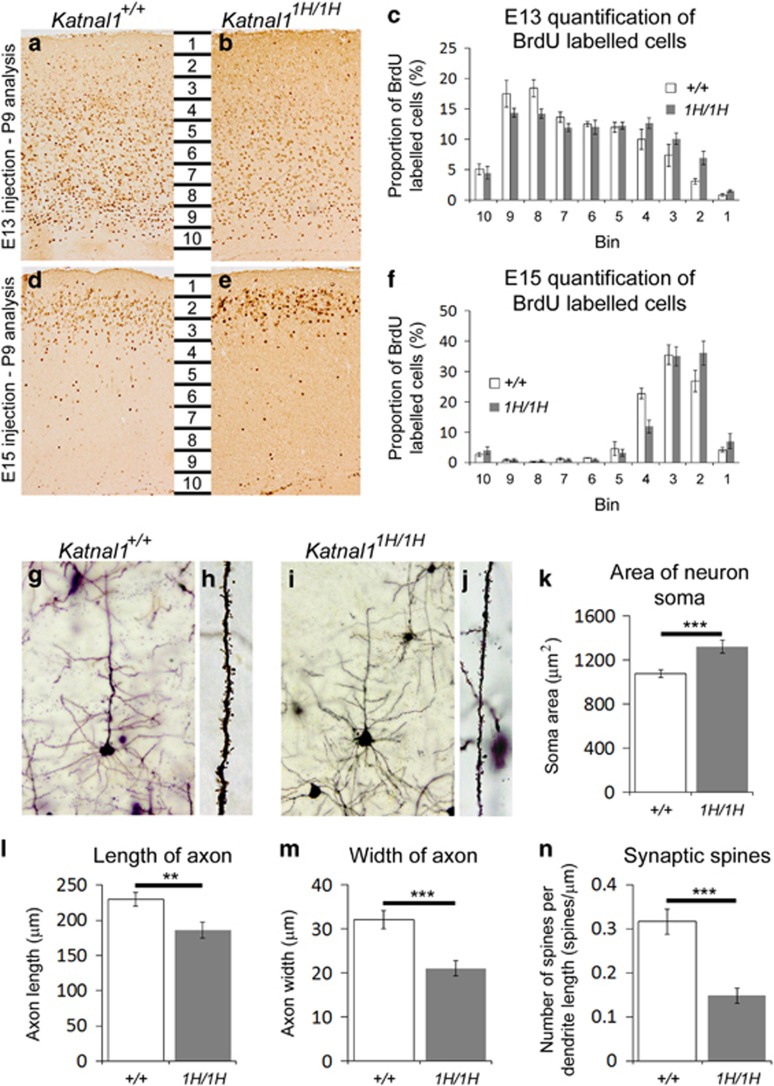
Neuronal migration and morphology abnormalities in *Katnal1*^*1H/1H*^ mice. (**a**,**b**,**d**,**e**): BrdU labelling of cortical neurons following injection of BrdU at either E13 (**a**,**b**) or E15 (**d**,**e**) in wild-type (**a**,**d**) and *Katnal1*^*1H/1H*^ (**b**,**e**) animals. Quantification of BrdU immunohistochemistry shows that *Katnal1*^*1H/1H*^ animals have increased numbers of labelled neurons closer to the cortical surface following injection at both E13 (**c**) and E15 (**f**). (**g**–**j**): Golgi labelling of neurons and dendrites in wild-type (**g**,**h**) and *Katnal1*^*1H/1H*^ (**i**,**j**) animals. (**k**–**n**): Quantification of golgi labelling shows that *Katnal1*^*1H/1H*^ animals have larger soma (**k**), shorter axons (**l**), thinner axons (**m**) and fewer dendritic spines (**n**) than wild types. **P⩽0.01; ***P⩽0.001.

The histological phenotypes of *Katnal1*^*1H/1H*^ mouse brains described above are suggestive of neuronal migration defects.^18^ We therefore investigated whether *Katnal1*^*1H/1H*^ mice showed abnormal neuronal migration using BrdU labelling of E13 and E15 embryos and quantified labelled cells in the cortex of P9 pups (described in reference 18). At both ages *Katnal1*^*1H/1H*^ animals had greater numbers of labelled neurons in bins close to the cortical surface (two-way ANOVA, interaction factors ‘genotype X bin’: E13 brains; F(9,80)=2.361, *P*=0.02. E15 brains; F(9,70)=2.412, *P*=0.019; Figures 4a–f). These results demonstrate a defect in neuronal migration during development, with the final location of *Katnal1*^*1H/1H*^ neurons positioned closer to the cortical surface compared to wild type. To confirm these results we used a Boyden chamber^19^ and performed *in vitro* neuronal migration analysis in E13.5 primary cortical neuronal cultures. Here we found that a greater proportion of *Katnal1*^*1H/1H*^ cortical neurons migrated to the base of the cell culture insert compared to wildtype controls (Supplementary Figure S3). Since in both BrdU labelling and the Boyden assay neurons from *Katnal1*^*1H/1H*^ animals migrated further than those of wild-type littermates, these results suggest that *Katnal1*^*1H/1H*^ cortical neurons show defects in the termination of cortical neuronal migration.

Given its role in cytoskeletal organisation, we also hypothesised that neuronal morphology is modulated by *Katnal1*. Analysis of golgi stained neurons from layers 2–3 of the cortex (Figures 4g and i) demonstrated that, compared to wild-type littermates, *Katnal1*^*1H/1H*^ neurons had larger soma (Figure 4k), and shorter and thinner axons (Figures 4l and m) (data and cohort details are given in Supplementary Table S3). Furthermore, analysis at higher magnification (Figures 4h and j), demonstrated that the number of synaptic spines on *Katnal1*^*1H/1H*^ neurons was significantly reduced compared to wild type (Figure 4n).

### *Katnal1*^*1H/1H*^ associated with defects in motile cilia

**Figure 5:**
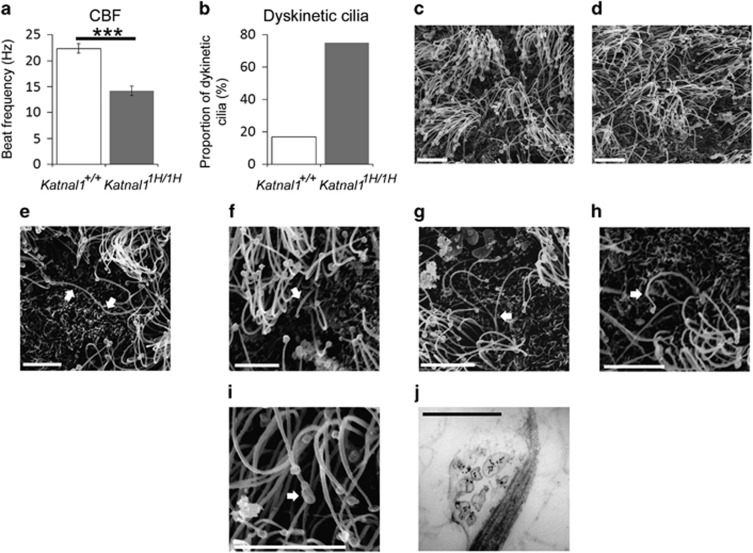
Ciliary dysfunction in *Katnal1*^*1H/1H*^ mice. (**a**,**b**): Ependymal motile cilia in the lateral ventricle of *Katnal1*^*1H/1H*^ animals have a significantly reduced CBF (**a**) and a higher proportion of ciliary dyskinesia (**b**) compared to wild-type littermates. (**c**,**d**): Scanning electron micrographs (SEM) of the ependymal motile cilia in the lateral ventricles of wiltype (**c**) and *Katnal1*^*1H/1H*^ (**d**) animals. (**e**–**i**): Structural ciliary abnormalities found in *Katnal1*^*1H/1H*^ animals include abnormally long cilia (**e**) abnormally short cilia (**f**) bifurcated cilia (**g**) kinks in the cilia (**h**) and swellings along the length of the cilia (**i**). Transmission Electron Micrographs show vesicular aggregates within ciliary swellings (**j**). Arrows in (**e**–**i**) indicate ciliary abnormalities. Scale bars: **c–i**=5 μm; ***j***, 500 nm (***P⩽0.001). CBF, ciliary beat frequency.

Recent studies have demonstrated that mutations in some microtubule severing enzymes can cause defects in cilia.^5^ Since such ciliary defects could underlie the phenotypes described above we studied the motile cilia of the ependymal lining of the lateral ventricle in sections of postnatal day 2 mouse brains from both *Katnal1*^*1H/1H*^ (*n*=4) and wild-type animals (*n*=3). We found that the ciliary beat frequency (CBF) of *Katnal1*^*1H/1H*^ animals was significantly attenuated compared to wild-type (CBF: wildtype=22.39±0.94 Hz, *Katnal1*^*1H/1H*^=14.25±0.92 Hz, *P*=0.0001; Figure 5a, Supplementary Movies S1). This reduction in CBF in *Katnal1*^*1H/1H*^ animals was also associated with an increased proportion of cilia with an abnormal beat pattern (ciliary dyskinesia) (proportion of dyskinetic cilia: wild type=17%, *Katnal1*^*1H/1H*^=75%) (Figure 5b and Supplementary Movies S1). Visual inspection of the cilia identified a number of ciliary abnormalities such as a swollen ciliary tip (Supplementary Movie S3) or extremely long cilia (Supplementary Movie S4) scattered throughout the field of cilia in *Katnal1*^*1H/1H*^ ventricles. These abnormalities were observed in approximately 25% of *Katnal1*^*1H/1H*^ brain slices. The abnormal cilia always showed a dyskinetic beat pattern and lower beat frequency. To further investigate ciliary morphology we performed scanning electron microscopy upon the ependymal lining of the lateral ventricles of both *Katnal1*^*1H/1H*^ (*n*=3) and wild-type animals (*n*=3; Figures 5c and d). Cilia measurements showed no significant differences in average cilia length between genotypes (average cilia length: wild type=6.22±0.86 μm, *Katnal1*^*1H/1H*^=6.54±0.94 Hz, *P*=0.303). However in *Katnal1*^*1H/1H*^ samples we noted the presence of both long and short cilia (Figures 5e and f; defined as two standard deviations longer or shorter than the average cilia length) that were not present in wild-type samples. In addition, inspection of *Katnal1*^*1H/1H*^ cilia identified ciliary abnormalities including bifurcated cilia (Figure 5g), abnormal kinks and bends in the cilia (Figure 5h) and swellings along the length of the cilia (Figure 5i). Transmission electron microscopy of ependymal cilia found that vesicular aggregates were present within the ciliary swellings described above (Figure 5j). Although these abnormalities were present in only a small proportion (<1%) of *Katnal1*^*1H/1H*^ cilia, they were notably absent from wild-type cilia.

## Discussion

Microtubule severing enzymes play diverse roles in the nervous system.^1, 2^ However, at present the microtubule severing enzyme *Katnal1* is poorly defined in the context of CNS development and function. Here we present a detailed phenotypic analysis of *Katnal1*^*1H*^ and show that the mutation is associated with changes in circadian rhythms, sleep and behaviour. Furthermore we demonstrate that defects in brain histopathology, neuronal migration and neuronal morphology underlie these phenotypes. Finally we also demonstrate that *Katnal1*^*1H*^ causes a range of defects in the motile cilia of ventricular ependymal cells. The data we present here are the first to associate KATNAL1 with such dysfunctions with important implications for clinical association studies.

The *Katnal1*^*1H*^ mutation was initially identified with a circadian deficit including a short free-running period and advanced activity onset. However subsequent *ex vivo* experiments using SCN slices of animals carrying the PER2::LUC reporter gene demonstrated no defects in SCN cellular rhythms, suggesting that the core circadian clock was unperturbed by the mutation. Phenotypes in circadian running wheel rhythms that are not associated with changes to the core clock mechanism have also been reported in mouse models of schizophrenia.^25^ Here it has been suggested that the wheel running changes observed are the result in defects in output pathways from the SCN circadian clock. Similarly, in *Katnal1*^*1H/1H*^ mice we hypothesise that the defects we demonstrate in neuronal anatomy and neuronal morphology may disrupt output signals from the SCN. Alternatively given that various neuropeptides such as oxytocin are secreted in a circadian manner from ependymal cells lining the third ventricle of the brain,^26^ altered ventricular morphology and ciliary function in *Katnal1*^*1H/1H*^ mice may disrupt the circulation of factors secreted by the ciliated ventricular ependymal cells and contribute to the disruption of the behavioural rhythms observed.

The behavioural consequences of microtubule severing enzyme dysfunction in mouse models have been poorly characterised. Currently the phenotypes described are limited to motor dysfunction in mice lacking the *Spg4* gene^27^ and head shaking and circling in the *Fign* mutant.^7, 28, 29^ In contrast, here we demonstrate that loss of function of Katnal1 is associated with a range of behavioural phenotypes, including changes in circadian activity, poor learning and memory, hyperactivity in a novel environment (the open field) and deficits in USVs. Notably the learning and memory, anxiety and vocalisation phenotypes reprise the clinical symptoms of ID, increased anxiety in novel situations and delays in language acquisition reported in human patients who carry microdeletions incorporating haploinsufficiency of *KATNAL1*.^8, 9^ While it is also worth noting that mutant mice spend more time the centre of the open field than wild types (implying that *Katnal1*^*1H/1H*^ animals show reduced anxiety), we suggest that this result is confounded by the hyperactivity in novel environments phenotype we also describe in mutant mice. This observation is backed up by the fact that mutant animals showed increased activity in all regions of the open field rather than just the anxiolytic periphery. Here we also highlight defects in *Katnal1*^*1H/1H*^ mice such as compromised neuronal migration and morphology which may underpin such phenotypes. In *Drosophila*, the homologue of *Katnal1* (*kat-60L1*) has been demonstrated to play a critical role in neuronal morphology during development,^30^ however the data that we present here is the first to demonstrate a similar phenotype in mammals and furthermore suggests how subtle perturbations to KATNAL1 function may contribute to specific neural and behavioural conditions. For example, defects in neuronal migration, synaptic spines and neuronal morphology such as those we have demonstrated here, have been suggested to underpin ID in conditions such as lissencephaly,^18^ Down’s syndrome^31^ and Rett syndrome.^32^ While we are not suggesting that *Katnal1* is causative for these conditions, similarities in symptoms and neuronal phenotypes between these conditions and those linked to *Katnal1* dysfunction should be appreciated. Furthermore a rare mutation in *KATNAL1* has been associated with schizophrenia^10^ (http://atgu.mgh.harvard.edu/~spurcell/genebook/genebook.cgi?user=guest&cmd=verb-gene&tbox=KATNAL1) and KATNAL1 has been shown to interact with the schizophrenia associated gene DISC1.^33^ In line with these observations we note that increases in ventricular volume and reductions in synaptic spines have been reported in schizophrenic patients^34, 35^ and our data demonstrates the same phenotypes in *Katnal1*^*1H/1H*^ mice. Thus the range of phenotypes associated with defects in the function of *Katnal1* strongly suggests that the gene should be considered in the pathology of disorders such as ID and schizophrenia.

We do note one key genetic difference between the human patients and *Katnal1*^*1H/1H*^ animals. While the human patients were all heterozygous for the *Katnal1* deletion, we found no phenotype in heterozygous mutant mice (data not shown) suggesting that while haploinsufficiency is causative for phenotypes in humans, mice require complete loss of KATNAL1 function to show similar effects. A similar discrepancy between humans and mice has also been noted for the intellectual disability candidate gene *CTNNB1.*^17^ While heterozygous loss of function mutations in *CTNNB1* are causative for intellectual disability in humans, conditional knock outs for *CTNNB1* have no reported behavioural or craniofacial phenotypes.^36, 37^ These differences demonstrate that while mouse models of intellectual disability are of great use in our understanding of the causative mechanisms which underlie the condition, there are still genetic and neurodevelopmental differences between species which also must be taken into account. We also note that while the *Katnal1*^*1H*^ mutation shows a loss of catalytic function in both HEK293 cells and Sertoli cells,^23^ this loss of function has not been verified in neuronal cells. However, given that our data demonstrates that the *Katnal1*^*1H*^ mutation lies in an essential catalytic domain and that we show neuronal phenotypes in *Katnal1*^*1H/1H*^ mice, we would expect to see the same loss of catalytic function in neurons.

The data we present here also demonstrate defects in motile cilia in *Katnal1*^*1H/1H*^ mice. Ciliary disruptions in humans (ciliopathies) include Bardet-Biedl and Joubert syndrome.^38^ While there is currently limited data available regarding the behavioural phenotypes of mouse models of ciliopathies, we note that ciliary dysfunction in mice has been linked with learning and memory^39^ and vocalisation phenotypes,^40^ both of which were disturbed in the *Katnal1*^*1H/1H*^ mice described here. It is also notable that the neuronal migration and enlarged ventricle phenotypes that we describe in *Katnal1*^*1H/1H*^ mice recapitulate features associated with known ciliopathy gene mutations.^41, 42, 43, 44^ Furthermore in Bardet-Biedl syndrome mouse models ciliary defects such as reduced CBF^45^ and structural defects such as abnormal lengthening and swellings along their length^41^ have been described, that are similar to those we describe in *Katnal1*^*1H/1H*^ mice. There is strong evidence that ciliopathy associated genes play a number of roles in neuronal development by affecting processes such as progenitor proliferation or maintenance of the radial glia scaffold.^43^ However it is also clear that defects in microtubule organisation also affect synaptic structure.^2^ At present it is difficult to disentangle the relative contributions of defects in microtubule severing and ciliary abnormalities to the overall phenotypes we observe in *Katnal1*^*1H/1H*^ mice. Further investigations are required to clarify the impacts of these two processes. However it is notable that while defects in cilia structure may contribute to the phenotypes we describe in *Katnal1*^*1H/1H*^ mice, they are far less prominent in *Katnal1*^*1H/1H*^ mice than in other mouse ciliopathy models,^41^ suggesting that the ciliary component of KATNAL1 dysfunction may be mild compared to other ciliopathies. Similarly while hydrocephalus has been suggested to be a component of some ciliopathy mouse models,^46^ *Katnal1*^*1H/1H*^ mice showed only increased ventricle size rather than an increased incidence of hydrocephalus, further suggesting the ciliary defects in these animals are mild compared to other ciliopathies.

In summary the data presented here clearly demonstrate that KATNAL1 plays an important role in a variety of neuronal processes including neuronal migration, neuronal morphology and ependymal ciliary function. The downstream effect of these defects leads in turn to a number of behavioural changes including in learning and memory, reaction to anxiogenic situations and circadian rhythms. These data therefore highlight how perturbations in KATNAL1 may play a role in neuronal dysfunction and demonstrates that the enzyme is a novel candidate in the study of behavioural and neurodevelopmental disorders.

## Supplementary Material

Supplementary Information

Supplementary Figure s1

Supplementary Figure s2

Supplementary Figure s3

Supplementary Movie s1

Supplementary Movie s2

Supplementary Movie s3

Supplementary Movie s4
