# Supplementary material for: A missense mutation in Katnal1 underlies behavioural, neurological and ciliary anomalies
Source: Mol Psychiatry. 2017 Apr 4;23(3):713–22. doi: 10.1038/mp.2017.54 (PMC5761721; doi:10.1038/mp.2017.54)
Supplement: Supplementary Information [file mp201754x1.docx]

**Supplementary information.**

**Methods.**

3D modelling of protein structure.

3D modelling of protein structure was performed as previously described.^S1^ Briefly, using PyMOL (<http://www.pymol.org/>) we modelled the structure of the AAA domain of KATNAL1 by overlaying the previously reported x-ray model of the *Drosophila* SPASTIN C-terminal^S2^ with the amino acid sequence of the C-terminal of mouse KATNAL1.

Circadian wheel running.

Mice were singly housed in cages containing running wheels, placed in light controlled chambers and wheel running activity monitored via ClockLab (Actimetrics). Animals were monitored for seven days in a 12 hour light/dark cycle (100 lux light intensity), twelve days in constant darkness and twelve days in constant light .

Ex vivo SCN slice recordings.

Organotypic SCN slices were prepared as described^S3^ from P10- P11 pups, by dissection of the hypothalamus using a McIlwain Tissue Chopper, and placing the coronally sliced SCN on Millipore tissue culture inserts placed in a 35mm Petri dish with culture medium. The slices were allowed to settle onto the insert for at least 1 week at 37 degrees in a CO2-gassed, humidified

incubator. They were then transferred to medium with luciferin for bioluminescent recording and the dish sealed with a glass cover slip and placed under a photomultiplier tube to record circadian cycles of PER2::LUCIFERASE -dependent bioluminescence in a light-tight incubator at 37 degrees.

Sleep assessment by electroencephalography and electromyography.

Mice were anesthetized with Ketamine/Xylazine, 90-150 K/ 7.5-16 X, IP and implanted with an EEG/EMG transmitter (Data Sciences, F20-EET, Gold system). Sleep deprivation was achieved by gentle handling of mice for 6 hours. EEG activity was sampled at 500 Hz with a filter cut-off of 50 Hz and was acquired using Dataquest A.R.T. software (DSI).

Behavioural phenotyping.

*Spontaneous alternation*: Mice were placed in a walled T-maze (black polyvinyl chloride, lined with sawdust; stem = 88cm X 13cm; arms = 32cm X 13 cm) and allowed to enter an arm of their choice. The mouse was confined in the goal arm for 30 seconds, before being allowed a second free choice of arm. An alternation was recorded if the second choice differed from that of the first. One trial was performed per day for 10 days.

*Morris water maze*: Acquisition trials were performed for six days in which mice were subjected to four trials with a 10 to 15 minute inter-trial period. Animals were monitored by EthoVision XT analysis software (Noldus).

*Video tracking in the home cage:* Mice were singly housed and placed in light controlled chambers with NIR miniature CCD cameras positioned above the cages (Maplin, UK). Monitoring during dark periods was performed using infrared illumination. Video monitoring was performed for a 24 hour period over a 12 hour light/dark cycle (100 lux light intensity). Video files were uploaded to ANYmaze video analysis software (Stoetling) for activity analysis.

*Open field behaviour*: Mice were placed into a walled arena (grey polyvinyl chloride; 45cm X 45cm) and allowed to explore for 20 minutes. Animals were monitored by EthoVision XT software.

*Ultrasonic vocalisation*: 7-8 day old pups were placed in a cage containing a microphone sensitive to ultrasonic sounds. USVs were recorded with a sampling rate of 250 kHz for 5 minutes and analysed by with SASLab Pro (Version 4.40) (Avisoft).

Immunofluorescence.

For calbindin and VIP immunofluorescence brains (4 animals per genotype; females; 4months old) were immersion fixed in 4% paraformaldehyde for 4 hours before cryopreservation in 20% sucrose (in PBS) overnight at 4^o^C. Brains were then mounted in OCT and 30μm sections taken using a cryostat and placed, free floating, into PBS. Sections were blocked in 2% goat serum before overnight incubation with primary antibody (either rabbit anti-calbindin or rabbit anti-VIP primary antibodies (Abcam; ab11426 and ab43841 respectively) at 1:200 dilution). Following this, sections were washed in PBS before incubation in alexa-flor 488 conjugated donkey anti-rabbit secondary antibody (Thermo Fisher Scientific; A11070).

For CUX1, FOXP2 and CTGF immunofluorescence, mice (3 animals per genotype; females; 6months old) were transcardially perfused with 4% PFA, the brains dissected and postfixed overnight in 4% PFA at 4^o^C. 50μm sections were taken using a vibrating microtome and free floating immunolabelling was performed as described for calbindin and VIP above, using 5% donkey serum, 3% BSA and 0.2% triton-X100 to block sections for FOXP2 and CTGF immunofluorescence and 10% donkey serum, 3% BSA and 0.2% triton-X100 to block sections for CUX1 immunofluorescence. The primary antibodies used were rabbit anti-CUX1 (Santa Cruz (sc-13024), at 1:50 dilution), rabbit anti-FOXP2 (Abcam (ab16046), at 1:4000 dilution) and goat anti-CTGF (Santo Cruz (sc-14939), at 1:1000 dilution). The secondary antibodies used were alexa-flor 488 conjugated donkey anti-goat and alexa-flor 568 conjugated donkey anti-rabbit secondary antibodies (Thermo Fisher Scientific; A11055 and A10042 respectively). Sections were viewed on a Zeiss Axio-Examiner 710 NLO multi-photon microscope. Images were taken using Zen (Zeiss).

Image analysis was performed using ImageJ.^S4^

Neuronal migration assessment.

For *In vivo* neuronal migration assessment pregnant mothers were injected with BrdU when the embryos had reached either E13 or E15 (three mothers per age group). Brains were taken from the subsequent pups at P9 (n: E13: wildtype=7 *Katnal1^1H/1H^*=5; E15: wildtype=4 *Katnal1^1H/1H^*=6). Brains were sectioned and cortical sections immuno-labelled for BrdU using anti-BrdU primary antibody (Abcam, Ab6323, 1:100 dilution), biotinylated anti-rat secondary antibody (Vector, BA-9401, 1:50 dilution) and DAB substrate kit for peroxidase (Vector). The labelled cortices were imaged and cell migration was analysed by dividing the cortex into ten equal bins extending from the intermediate zone to the cortical surface and the number of cells in each bin. Cell counts were taken using ImageJ.

*In vitro* neuronal migration assessment was performed using a Boyden chamber migration protocol. The cortices of E13.5 embryos (n=3 of each genotype from two different litters) were dissected in neurobasal medium (Invitrogen) on ice, digested for 20 minutes with trypsin (0.25%) at 37°C, and washed several times. Cells were allowed to grow on a poly-D-lysine (Sigma-Aldrich) and laminin (Invitrogen) coated cell culture insert (8μm pore size) in complete neuronal growth medium (neurobasal medium containing 1% l-glutamine, 1% penicillin/streptomycin, and 2% B-27 supplement). The cell culture insert was placed in a 6 well cull culture dish in complete neuronal growth media. Following an overnight incubation, the media in the cell culture insert was replaced with neurobasal medium and fresh complete neuronal growth media placed into the cell culture dish. Following a 24 hour incubation, the cell culture insert was removed and the cells attached to the insert were fixed in 4% paraformaldehyde, and visualised using DAPI. The proportion of cells which had migrated onto the lower side of the cell culture insert was then calculated.

Micro–computed tomography (μCT) scanning.

Mice were perfused with 4% PFA under terminal anaesthesia. Brains were dissected and the cerebellum removed before immersion in 4% PFA for 4 days, immersion in 50% Lugols solution for 5 days, embedding in 1% agarose and scanning on a Skyscan 1172 at 90 kV, 112µA using an aluminium and copper filter, a rotation step of 0.250 degrees and a pixel size of 4.96µm.

Segmentation, volume calculation and 3D modelling was performed using ITK-SNAP version 3.0.0 and 3DSlicer.

Motile cilia analysis.

Brains from P2 mice (n: wildtype=4, *Katnal1^1H/1H^*=6; from 3 litters) were dissected, and the dorsal cerebral half was sectioned (250µm) through the floor of the lateral and 3rd ventricle using a vibrotome. Slices were mounted in a microscope chamber slide in pre-warmed (37^o^C) M199 medium (Life Technologies) and observed using a high-speed video camera (MotionPro X4). Ciliary beat frequency (CBF) was determined by timing a given number of individual cilia beat cycles (CBF in Hz = 500 (Number of frames per second)/5 (frames elapsed for 5 ciliary beat cycles) X 5 (conversion per beat cycle)). A dyskinetic cilium was scored when the beat pattern was abnormal.

Electron microscopy.

For Scanning Electron Microscopy, mouse brains were taken (n= 3 (1 male, 2 female)) per genotype; 9 months old) and the ependymal lining of lateral ventricle dissected. Samples were fixed overnight in 2.5% glutaraldhyde, 2% paraformaldehyde in 0.1M phosphate buffer at 4^o^C. Samples were then incubated in 2% osmium tetroxide for 1 hour, washed six times in water and dehydrated through increasing strength ethanol solutions. Samples were then critical point dried using an Emitech K850 (KM technologies limited), mounted on stubs using silver paint (Agar scientific) and sputter coated with platinum using a Qurom Q150R S sputter coater (Quorum Technologies). Ependymal cilia were visualised and measured using a JEOL LSM-6010 scanning electron microscope (Jeol Ltd).

For Transmission Electron Microscopy, the ependymal lining of the lateral ventricle of the mouse brains (n= 3 males per genotype) were brushed using dental sticks to collect ependymal cilia. Cilia samples were fixed in 2.5% glutaraldhyde in Sorenson phosphate buffer for 48 hours. Following this samples were postfixed in 1% osmium tetroxide, embedded in 2% agar, dehydrated and processed into resin blocks. Ultra-thin sections were cut at 70nm and sections were collected on 200 mesh thin bar copper grids. Sections were stained in 1% uranyl acetate and Reynolds lead citrate and examined by transmission electron microscopy.

Methodological and statistical considerations.

All animal behaviour, histological, cell migration and neuronal morphology studies were conducted blind to genotype although no randomisation was used. Power calculations previously conducted by our group were used to ensure that all animal cohorts were of adequate size. All data was checked to confirm that it met with the assumptions of the statistical tests used. Additionally data variance was compared between the groups analysed by analysis of the standard deviations between data groups. This was found to be similar in all cases with the exception of cilia length. However, as noted in the results section, this deviation in cilia length is likely to be due to a phenotype in the *Katnal1^1H/1H^* mouse line. No samples were excluded.

**Supplementary figure legends**

**Figure S1: Structural analysis of Katnal1^1H^ mutation. A:** Sequence alignment of the AAA domains of *Drosophila* SPASTIN, mouse KATNAL1 and the KATNAL1^1H^ mutation shows strong sequence conservation between the proteins. The locations of the five α helices of the nucleotide-binding domain (NBD) are marked as α1 to α5. The position of the KATNAL1^1H^ mutation is designated by *. Given the strong homology between the proteins we modelled the structure of the AAA domain of KATNAL1 by overlaying the previously reported x-ray model of the *Drosophila* SPASTIN C-terminal^S5^ with the amino acid sequence of the C-terminal of mouse KATNAL1. **B:** The KATNAL1^1H^ mutation (blue) lies in close proximity to Leu567 of *Drosophila* SPASTIN (yellow). Mutations in Leu567 are associated with hereditary spastic paraplegias.^S6^ **C**: Superposition of mouse KATNAL1 (cyan) with *Drosophila* SPASTIN (orange), showing the α1/α2 connecting loop (purple) and hydrophobic interactions between α1 and α4. **D**: Close view of the hydrophobic interactions between α1 and α4 (hydrophobic residues shown in light green). In wildtype KATNAL1 the Leu residue (blue) conserves the hydrophobic interaction. In KATNAL1^1H^, the mutant Val residue (red) is spatially distant from the NBD, breaking the hydrophobic bond.

**Figure S2: Core clock functions in *Katnal1^1H/1H^* mice.** Recordings of the PER2::LUC reporter in SCN slices reveal no difference in the period (**A**) or the robustness (**B**) of bioluminescence rhythms between *Katnal1^1H/1H^* and wildtype control SCN.

**Figure S3: Boyden assay of neuronal migration in *Katnal1^1H/1H^* primary neurons.** Primary cortical neurons from *Katnal1^1H/1H^* and wildtype littermate E13.5 embryos were seeded on the upper side of a cell culture membrane in minimal culture media. The well below the membrane contained complete cell culture media. Following a 24 hour incubation the membrane was removed, fixed and stained with DAPI. The cells on the lower side of the membrane (cells which migrated through from the upper side of the membrane) were then counted and expressed as a proportion of the total number of seeded cells. For further details of the technique, please see reference S14. This assay demonstrated that a greater proportion of *Katnal1^1H/1H^* primary neurons migrated to the lower side of the membrane compared to wildtype (proportion of cells on the base of insert: wildtype = 5.73 ±0.61%, *Katnal1^1H/1H^* = 9.54 ±0.42%, p=0.0004). We note that these results are similar to those seen in Nrn1 overexpressing neurons, which show enhanced neuronal migration.^S7^

**Supplementary movie files.**

**Movie S1: Beating ependymal cilia from a wildtype brain slice.** Motile cilia of the ependymal lining of the lateral ventricle from a section of postnatal day 2 wildtype mouse brain.

**Movie S2: Beating ependymal cilia from a *Katnal1^1H/1H^* brain slice.** Motile cilia of the ependymal lining of the lateral ventricle from a section of postnatal day 2 *Katnal1^1H/1H^* mouse brain.

**Movie S3: Cilia with a swollen tip from a *Katnal1^1H/1H^* brain slice.** A small proportion of cilia in *Katnal1^1H/1H^* ependymal sections show a number of ciliary abnormalities including swellings at the cilia tips.

**Movie S4: Extremely long cilia from a *Katnal1^1H/1H^* brain slice.** A small proportion of cilia in *Katnal1^1H/1H^* ependymal sections show a number of ciliary abnormalities including extremely long cilia.

**SUPPLEMENTAL DATA TABLES**

**Table S1:** Circadian and sleep phenotype data and cohort details. Light condition definiations: LD = 12/12 light/dark cycles; DD = Constant darkness; LL = Constant light.Data are presented as mean ±SEM (standard error of the mean).

| **Phenotyping test** | **Animal cohort** | **Measure** | **Light condition** | **Genotype** | | **p** |
| --- | --- | --- | --- | --- | --- | --- |
|  |  |  |  | ***Katnal1^+/+^*** | ***Katnal1^1H/1H^*** |  |
| Circadian wheel running | 11 *Katnal1^1H/1H^* and 10 wildtype littermates (females, 3 months old, from 9 different litters) | Circadian period (hours) | DD | 23.351 ±0.106 | 22.98 ±0.069 | **0.00966** |
|  |  |  | LL | 25.287 ±0.124 | 25.386 ±0.138 | 0.59820 |
|  |  | Activity (wheel rotations/day) | Light phase of LD | 317.775 ±56.72 | 681.829 ±142.97 | **0.03434** |
|  |  |  | Dark phase of LD | 9989.1 ±1124.46 | 12331.557 ±1071.38 | 0.14793 |
|  |  |  | Total LD | 10306.875 ±1127.31 | 13013.386 ±1078.51 | 0.09902 |
|  |  |  | DD | 16588.368 ±2102.86 | 18108.916 ±1746.23 | 0.58193 |
|  |  |  | LL | 9452.016 ±1076.51 | 9752.073 ±1857.87 | 0.89319 |
|  |  | Proportion of activity in light phase (%) | LD | 3.3551 ±0.597 | 5.496 ±1.176 | 0.13201 |
|  |  | Length of active phase (hours) | LD | 9.004 ±0.215 | 9.238 ±0.195 | 0.42995 |
|  |  |  | DD | 10.815 ±0.439 | 10.173 ±0.49 | 0.34130 |
|  |  |  | LL | 13.509 ±1.087 | 12.651 ±1.24 | 0.60943 |
|  |  | Chi squared amplitude | LD | 1186.591 ±49.42 | 1251.33 ±85.29 | 0.46442 |
|  |  |  | DD | 1693.396 ±71.05 | 1602.073 ±112.25 | 0.52521 |
|  |  |  | LL | 1272.353 ±82.63 | 1184.327 ±159.7 | 0.63159 |
|  |  | Onset of activity relative to lights off (min) | LD | -5.8 ±0.869 | -11.818 ±0.046 | **0.04578** |
|  |  | Onset of activity on release into DD relative to LD (min) | LD/DD | -41.8 ±5.403 | -124.272 ±13.402 | **0.00002** |
| *Per2:Luciferase* bioluminescence rhythms | 7 *Katnal1^1H/1H^* and 5 wildtype SCN slices, from 3 different litters | Bioluminescence rhythm period (hours) | N.A. | 24.4 ±0.138 | 24.4 ±0.104 | 0.80542 |
|  |  | Robustness (relative amplitude error) | N.A. | 53.7 ±2.95 | 55.9 ±2.06 | 0.53835 |
| Baseline EEG sleep | 5 *Katnal1^1H/1H^* and 5 wildtype littermates (females, 5 months old, from 5 different litters) | Total time spent asleep (min) | Light phase of LD | 409.54 ±12.4 | 417.02 ±15 | >0.05 |
|  |  |  | Dark phase of LD | 109.4 ±27.4 | 154.24 ±13.4 | >0.05 |
|  |  | Total time in REM sleep (min) | Light phase of LD | 48.86 ±2.92 | 52.68 ±1.98 | >0.05 |
|  |  |  | Dark phase of LD | 9.4 ±2.18 | 12.9 ±2.29 | >0.05 |
|  |  | Total time in non-REM sleep (min) | Light phase of LD | 360.68 ±10.7 | 364.34 ±15.5 | >0.05 |
|  |  |  | Dark phase of LD | 100.02 ±25.5 | 141.3 ±12.5 | >0.05 |
| EEG sleep following sleep deprivation (SD) |  | Total time spent asleep (min) | 6 hours light following SD | 242.42 ±9.6 | 251.84 ±12.7 | >0.05 |
|  |  |  | 12 hours dark following SD | 218 ±13.4 | 192.2 ±15.6 | >0.05 |
|  |  | Total time in REM sleep (min) | 6 hours light following SD | 29 ±2.8 | 35.4 ±2.7 | >0.05 |
|  |  |  | 12 hours dark following SD | 21.94 ±2.8 | 18.92 ±1.9 | >0.05 |
|  |  | Total time in non-REM sleep (min) | 6 hours light following SD | 213.4 ±7.5 | 216.4 ±10 | >0.05 |
|  |  |  | 12 hours dark following SD | 196.06 ±13.4 | 173.26 ±13.9 | >0.05 |

Table S2. Behavioural phenotyping data and cohort details. Data are presented as mean ±SEM (standard error of the mean).

| **Phenotyping test** | **Animal cohort** | **Measure** | | **Genotype** | | **p** |
| --- | --- | --- | --- | --- | --- | --- |
|  |  |  |  | ***Katnal1^+/+^*** | ***Katnal1^1H/1H^*** |  |
| Open field | 11 *Katnal1^1H/1H^* and 12 wildtype littermates (males, 4 months old, from 9 different litters) | Proportion of time spent in centre (%) | | 13.93 ±1.6 | 20.11 ±2.1 | **0.028** |
|  |  | Latency to enter centre (seconds) | | 116.76 ±14.11 | 78.8 ±23.15 | **0.035** |
|  |  | Distance moved (meters) | | 56.94 ±0.32 | 79.27 ±0.56 | **0.002** |
|  |  | Average velocity (cm/s) | | 4.74 ±0.27 | 6.62 ±0.47 | **0.002** |
| Ultrasonic vocalisation production | 6 *Katnal1^1H/1H^* and 6 wildtype littermates (pups, P7-8), from 6 different litters | Number of vocalisations | | 416.3 ±59 | 190.5 ±54 | **0.017** |
|  |  | Duration of vocalisations (seconds) | | 0.037 ±0.001 | 0.026 ±0.001 | **0.001** |
|  |  | Number of phrases during vocalisations | | 48 ±4 | 29 ±6 | **0.02** |
| Spontaneous alternation | 11 *Katnal1^1H/1H^* and 12 wildtype littermates (males, 4 months old, from 9 different litters) | Proportion of correct alternations (%) | | 80.83 ±3.13 | 56 ±6.7 | **0.0019** |
| Morris water maze | 8 *Katnal1^1H/1H^* and 8 wildtype littermates (females, 6 months old, from 5 different litters) | Time to find platform (seconds) | Day 1 | 27.5 ±4.9 | 46.9 ±3.8 | **<0.05** |
|  |  |  | Day 2 | 21.9 ±1.6 | 37.4 ±2.4 |  |
|  |  |  | Day 3 | 23.8 ±1.7 | 51.1 ±1.7 |  |
|  |  |  | Day 4 | 15.4 ±3.4 | 42.6 ±3.2 |  |
|  |  |  | Day 5 | 14.54 ±2.2 | 35.9 ±2.7 |  |
|  |  |  | Day 6 | 12.9 ±1.1 | 39.2 ±3.5 |  |
|  |  | Distance moved (meters) | Day 1 | 0.92 ±0.15 | 1.46 ±0.15 | **<0.05** |
|  |  |  | Day 2 | 0.78 ±0.06 | 1.06 ±0.09 |  |
|  |  |  | Day 3 | 0.89 ±0.11 | 1.48 ±0.1 |  |
|  |  |  | Day 4 | 0.49 ±0.11 | 1.1 ±0.12 |  |
|  |  |  | Day 5 | 0.44 ±0.08 | 0.98 ±0.12 |  |
|  |  |  | Day 6 | 0.37 ±0.05 | 1.21 ±0.09 |  |
|  |  | Time spent in quadrant on final day (seconds) | North-West | 12.9 ±1.4 | 15.4 ±2.6 | >0.05 |
|  |  |  | South-East | 10.7 ±3.2 | 12.13 ±2.9 | >0.05 |
|  |  |  | North-East | 3.1 ±1.9 | 10.31 ±3.2 | **<0.05** |
|  |  |  | South-West (correct quadrant) | 31.8 ±1.6 | 20.89 ±2.8 | **<0.05** |
|  |  | Average swim speed | | 0.0328 ±0.002 | 0.0287 ±0.001 | 0.055 |
|  |  | Improvement in finding platform (difference between latency on day 1 (100%) and latency on day 6) (%)) | | 52.9 ±6 | 16.6 ±7.3 | **<0.05** |
|  |  | Time to find platform in reverse trials (seconds) | Day 1 | 60 ±1 | 48.6 ±3 | >0.05 |
|  |  |  | Day 2 | 50.4 ±5 | 54.47 ±5 |  |
|  |  |  | Day 3 | 32.56 ±12 | 50.97 ±8 |  |
|  |  |  | Day 4 | 45.6 ±6 | 38.23 ±6 |  |
|  |  | Distance moved in reverse trials (meters) | Day 1 | 1.8 ±0.11 | 1.6 ±0.2 | >0.05 |
|  |  |  | Day 2 | 1.2 ±0.23 | 1.6 ±0.21 |  |
|  |  |  | Day 3 | 1 ±0.4 | 1.3 ±0.18 |  |
|  |  |  | Day 4 | 1.1 ±0.2 | 0.9 ±0.16 |  |

Table S3. Brain histology and neuronal morphology data and cohort details. Data are presented as mean ±SEM (standard error of the mean).

| **Technique** | **Cohort details** | **Measure** | | **Genotype** | | **p** |
| --- | --- | --- | --- | --- | --- | --- |
|  |  |  |  | ***Katnal1^+/+^*** | ***Katnal1^1H/1H^*** |  |
| Haematoxylin and eosin | 5 *Katnal1^1H/1H^* and 5 wildtype littermates (males, 5 months old, from 4 different litters) | Cell counts in hippocampal sections (cells/mm^2^) | CA1 | 0.007 ±0.0002 | 0.005 ±0.0004 | **0.0002** |
|  |  |  | CA3 | 0.006 ±0.0002 | 0.005 ±0.0002 | **0.0002** |
|  |  | Depth of cortical layer (% of total cortex size) | Layer 1 | 9.91 ±0.38 | 6.27 ±0.56 | **0.000006** |
|  |  |  | Layer 2/3 | 23.64 ±0.83 | 24.75 ±0.59 | 0.142 |
|  |  |  | Layer 4 | 10.74 ±0.42 | 10.39 ±0.67 | 0.535 |
|  |  |  | Layer 5 | 23.6 ±0.55 | 23.76 ±0.45 | 0.751 |
|  |  |  | Layer 6 | 32.11 ±0.55 | 34.84 ±0.62 | **0.0004** |
| μCT scanning | 4 *Katnal1^1H/1H^* and 4 wildtype littermates (males, 5 months old, from 5 different litters) | Ventricular volume (μm^3^) | | 4 ±0.25 | 11.5 ±0.84 | **0.0001** |
| Golgi staining | At least 10 neurons per mouse from 3 *Katnal1^1H/1H^* and 3 wildtype littermates (males, 6 months old, from 4 different litters) | Neuron soma area (μm^2^) | | 1077 ±35 | 1326 ±58 | **0.0002** |
|  |  | Length of axon (μm) | | 230 ±9.48 | 186 ±11.19 | **0.0039** |
|  |  | Thickness of axon (μm) | | 32.08 ±2.07 | 21.04 ±1.75 | **0.0002** |
|  |  | Number of axon branch points per μm | | 0.046 ±0.006 | 0.038 ±0.003 | 0.328 |
|  |  | Number of dendritic spines (spines per μm) | | 0.318 ±0.028 | 0.149 ±0.017 | **0.00001** |
|  |  | Distance from soma to 1st spine (μm) | | 50.48 ±5.92 | 50.13 ±10.12 | 0.977 |
|  |  | Number of primary dendrites per μm^2^ of soma | | 0.005 ±0.0002 | 0.005 ±0.0002 | 0.237 |
|  |  | Length of primary dendrites (μm) | | 58.61 ±5.71 | 57.01 ±7.21 | 0.667 |
|  |  | Number of primary dendrite branch points per μm | | 4.68 ±0.35 | 5.27 ±0.53 | 0.336 |
|  |  | Length of secondary dendrites (μm) | | 36.21 ±3.5 | 38.86 ±5.81 | 0.364 |

**Supplementary references.**

S1. Tucci, V., Kleefstra, T., Hardy, A., Heise, I., Maggi, S., Willemsen, M.H., *et al*. Dominant β-catenin mutations cause intellectual disability with recognizable syndromic features. J. Clin. Invest. 2014; 124: 1468-1482.

S2. Roll-Mecak, A., Vale, R.D., Structural basis of microtubule severing by the hereditary spastic paraplegia protein spastin. Nature. 2008; 451; 363-367.

S3. Hastings, M.H., Reddy, A.B., McMahon, D.G., Maywood, E.S., Analysis of circadian mechanisms in the suprachiasmatic nucleus by transgenesis and biolistic transfection. Methods Enzymol. 2005;393: 579-92.

S4. Abramoff, M.D., Magalhaes, P.J., Ram, S.J. 2004. Image Processing with ImageJ. Biophotonics Int. 11, 36-42.

S5. Roll-Mecak, A., Vale, R.D. Structural basis of microtubule severing by the hereditary spastic paraplegia protein spastin. Nature. 2008; 451: 363-367.

S6. Fonknechten, N., Marvel, D., Byrne, P., Davoine, C.S., Cruaud, C., Bonsch, D., et al. Spectrum of SPG4 mutations in autosomal dominant spastic paraplegia. Hum. Mol. Genet. 2000; 9: 637-644.

S7. Zito, A., Cartelli, D., Cappelletti, G., Cariboni, A., Andrews, W., Parnavelas, J., *et al*. Neuritin 1 promotes neuronal migration. Brain Struct. Funct. 2014: 219; 105-118
