# Supplementary figures and images for: A missense mutation in Katnal1 underlies behavioural, neurological and ciliary anomalies
Source: Mol Psychiatry. 2017 Apr 4;23(3):713–22. doi: 10.1038/mp.2017.54 (PMC5761721; doi:10.1038/mp.2017.54)

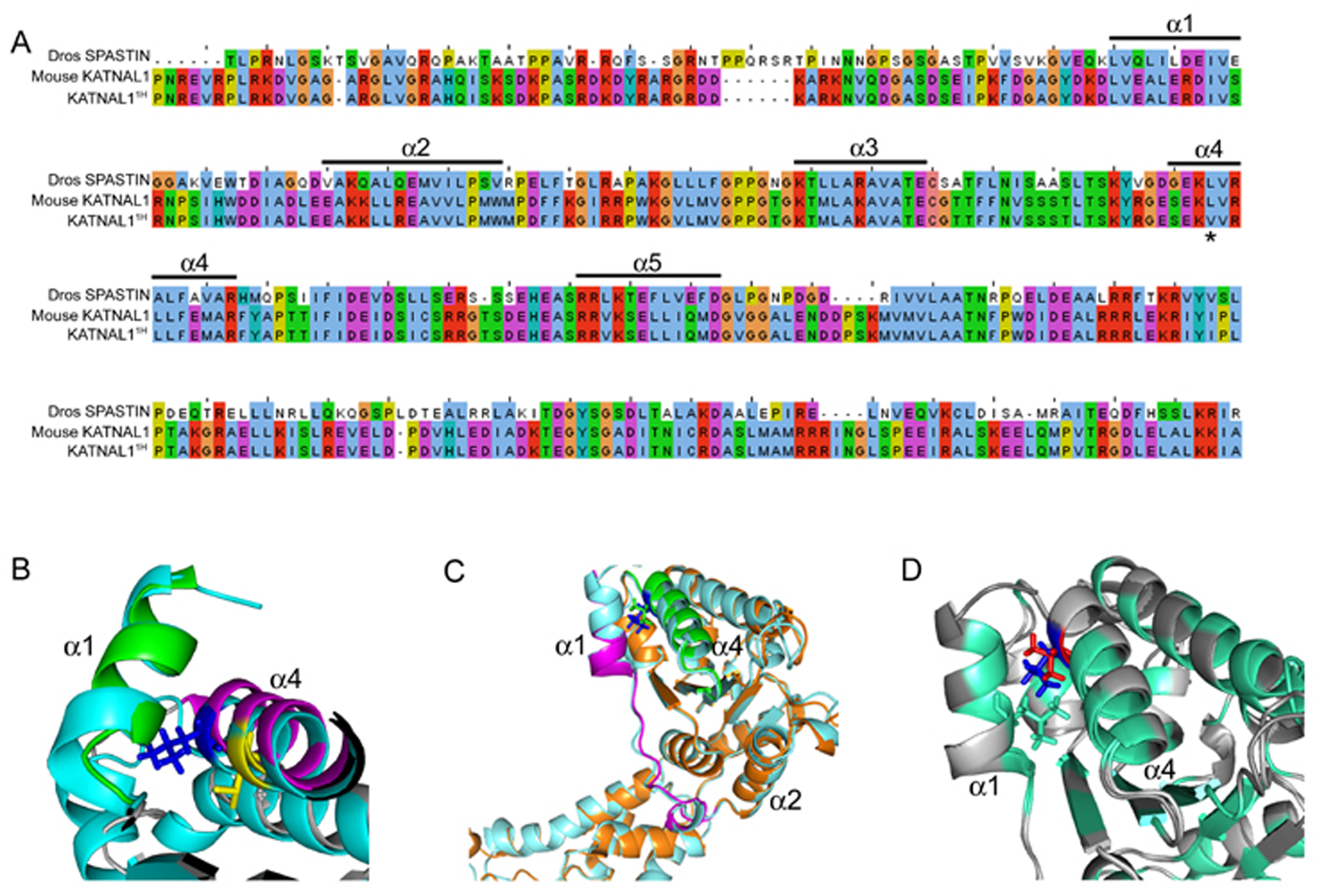

Supplement: Supplementary Figure s1 [file mp201754x2.tif]

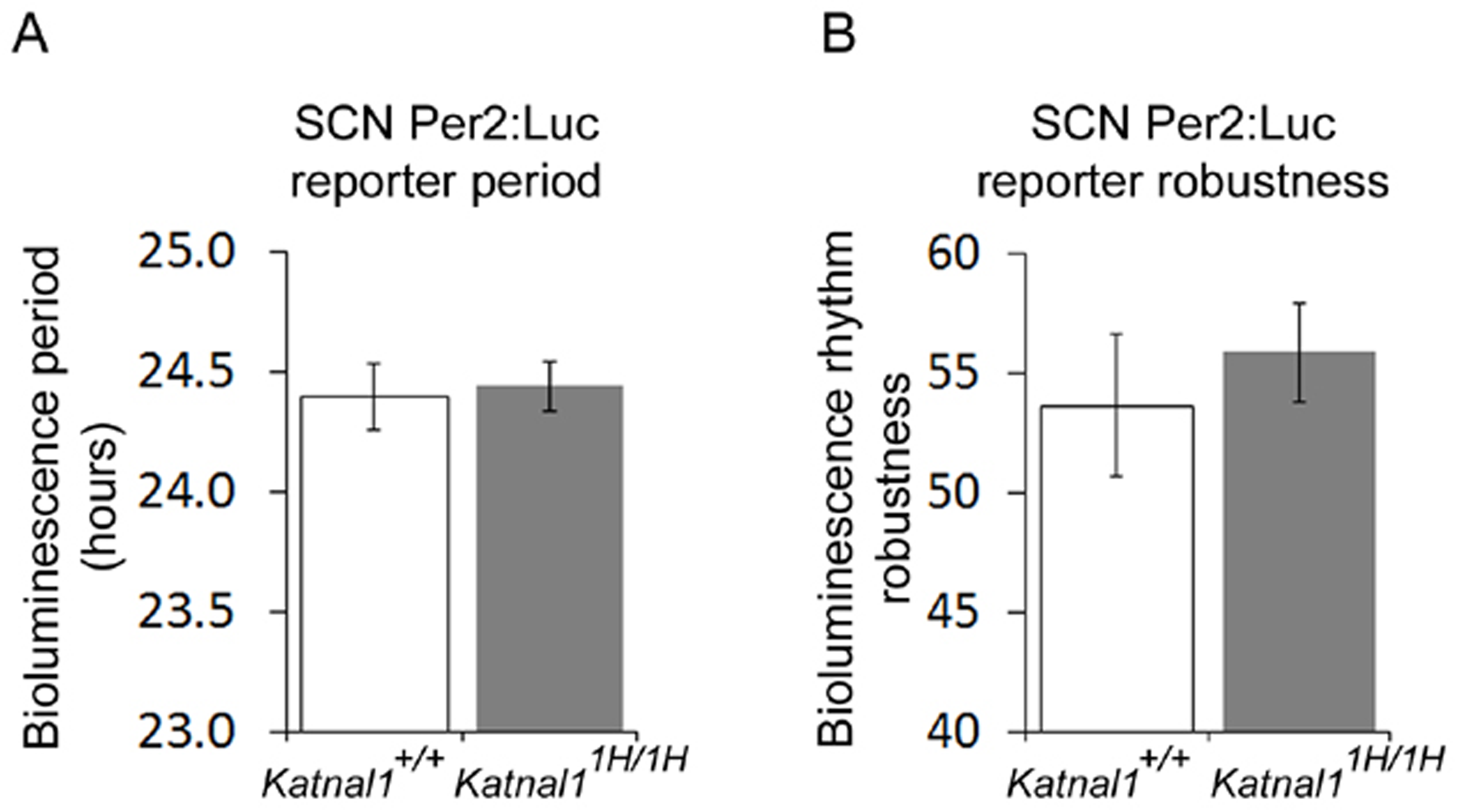

Supplement: Supplementary Figure s2 [file mp201754x3.tif]

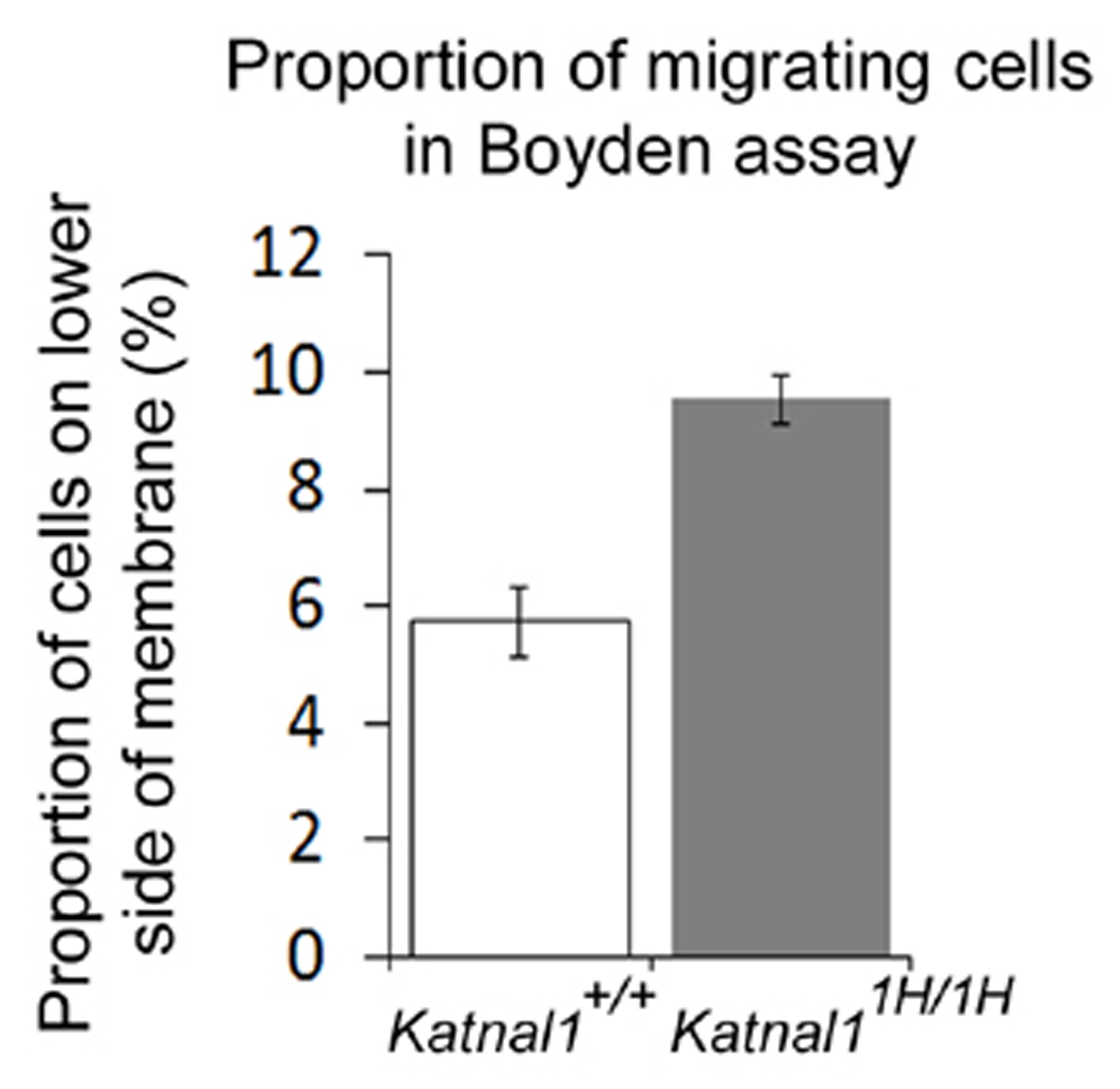

Supplement: Supplementary Figure s3 [file mp201754x4.tif]
